# Fast hit generation for discovery of inhibitors of DNA repair protein Pol eta

**DOI:** 10.1063/4.0000794

**Published:** 2025-10-27

**Authors:** Debanu Das

**Affiliations:** 1 XPose Therapeutics, San Carlos, CA, USA; 2 Accelero Biostructures, San Carlos, CA, USA

## Abstract

Polymerase eta (or Pol η or POLH) is a specialized DNA polymerase that can bypass certain blocking lesions in DNA replication, such as those generated by ultraviolet radiation (UVR) or cisplatin. POLH is deployed to replication foci for translesion (TLS) synthesis as part of the DNA damage response (DDR). POLH can bypass platinum-DNA adducts, negating benefits of treatment and enabling drug resistance in standard-of- care cancer therapies involving platinum-based clinical agents like cisplatin or oxaliplatin. POLH inhibition can sensitize cells to platinum-based chemotherapies. POLH has also been implicated in resistance to nucleoside analogs, such as gemcitabine. POLH overexpression has been linked to the development of chemoresistance in lung, ovarian and bladder cancers. Co-inhibition of POLH and the ATR serine/threonine kinase, another DDR protein, causes synthetic lethality (SL) in a range of cancers. All this reinforces that POLH is an emerging target for the development of novel oncology therapeutics.

Using a fragment-based drug discovery approach, we obtained first X-ray crystal structures of small novel drug-like compounds, i.e., fragments, bound to POLH, as starting points for the design of POLH inhibitors, to enable early drug discovery on POLH, which has been refractory to targeting by other methods. Our results can be quickly exploited in fragment growth and elaboration as well as analog scoping and scaffold hopping using medicinal and computational chemistry to advance hits to lead. An initial round of medicinal chemistry resulted in allosteric inhibitors with functional activity in an *in vitro* biochemical assay, leading to the rapid identification of an inhibitor to advance to subsequent rounds of chemistry to generate a lead compound. Crucially, our compound differs from the traditional nucleoside analog-based approaches for targeting DNA polymerases.

